# Sex-Specific Antioxidant and Anti-Inflammatory Protective Effects of AMPK in Cardiovascular Diseases

**DOI:** 10.3390/antiox14050615

**Published:** 2025-05-21

**Authors:** Lea Strohm, Dominika Mihalikova, Alexander Czarnowski, Zita Schwaibold, Andreas Daiber, Paul Stamm

**Affiliations:** 1 Department of Cardiology, Cardiology I, University Medical Center of the Johannes Gutenberg-University, 55131 Mainz, Germany; lstrohm@uni-mainz.de (L.S.); dominika.mihalikova@unimedizin-mainz.de (D.M.); aczarnow@students.uni-mainz.de (A.C.); zita.schwaibold@unimedizin-mainz.de (Z.S.); 2 German Center for Cardiovascular Research (DZHK), Partnersite Rhine-Main, 55131 Mainz, Germany

**Keywords:** AMPK, sex-specific differences, oxidative stress, antioxidant, cardiovascular diseases

## Abstract

Cardiovascular diseases such as coronary heart disease, heart failure, or stroke are the most common cause of death worldwide and are regularly based on risk factors like diabetes mellitus, hypertension, or obesity. At the same time, both diseases and risk factors are significantly influenced by sex hormones. In order to better understand this influence and also specifically improve the therapy of female patients, medical research has recently focused increasingly on gender-specific differences. The goal is to develop personalized, gender-specific therapy concepts for these diseases to further enhance health outcomes. The enzyme adenosine monophosphate-activated protein kinase (AMPK) is a central regulator of energy metabolism, protecting the cardiovascular system from energy depletion, thereby promoting vascular health and preventing cellular damage. AMPK confers cardioprotective effects by preventing endothelial and vascular dysfunction, and by controlling or regulating oxidative stress and inflammatory processes. For AMPK, sex-specific effects were reported, influencing metabolic and cardiovascular responses. Exercise and metabolic stress generally cause higher AMPK activity in males. At the same time, females exhibit protective mechanisms against insulin resistance or oxidative stress, particularly in conditions like obesity. Additionally, males subject to AMPK deficiency seem to experience greater cardiac and mitochondrial dysfunction. In contrast, females show improvement in cardiovascular function after pharmacological AMPK activation. These differences, influenced by hormones, body composition, and gene expression, highlight the potential to develop personalized, sex-specific AMPK-targeted therapeutic strategies for cardiovascular diseases in the future. Here, we discuss the most actual scientific background, focusing on the protective, gender-specific effects of AMPK, and highlight potential clinical applications.

## 1. Introduction

### Physiological Role and Cellular Distribution of AMPK

Cardiovascular diseases (CVDs) constitute the predominant cause of mortality on a global scale, accounting for more than 30% of all deaths worldwide in 2019 [1]. Most cardiovascular diseases are associated with oxidative stress, defined as an imbalance between the formation of reactive oxygen species (ROS), hydrogen peroxide and superoxide as primary species, and detoxification by antioxidant systems, disrupting redox signaling [2]. This imbalance can lead to various pathological conditions, including inflammation and vascular dysfunction, promoting the progression of cardiovascular diseases [3,4,5]. There is growing evidence that adenosine monophosphate-activated protein kinase (AMPK) plays a protective role in cardiovascular function by regulating endothelial function, inflammation, and redox homeostasis [6,7].

**Figure 1:**
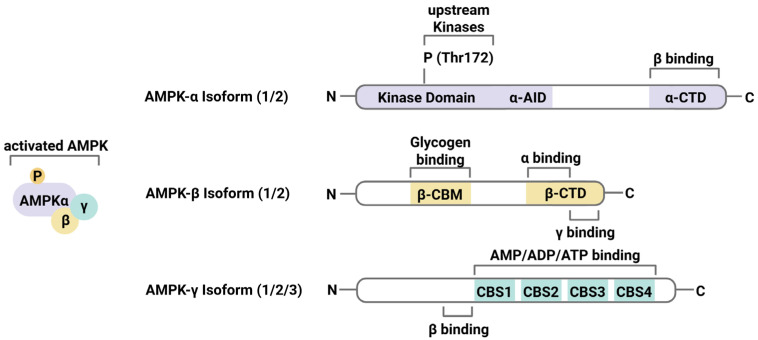
Schematic AMPK structure. AMPK is activated by the assembly of the three isoforms and phosphorylation of the kinase domain. Different binding domains in subunits: light purple, alpha subunit; light brown, beta subunit; light blue, gamma subunit. P = phosphorylation, Thr = threonine, AID = autoinhibitory domain, CTD = C-terminal binding domain, CBM = carbohydrate-binding module, CBS = cystathionine-β-synthase domain. The figure was created with BioRender.com and summarized from data in [17].

In eukaryotes, AMPK is ubiquitously expressed and a key regulator of cellular homeostasis, activated by energy stress or an increased adenosine monophosphate/adenosine triphosphate (AMP/ATP) ratio. The heterotrimeric serine/threonine protein kinase complex consists of an α-catalytic subunit and two regulatory subunits, β and γ. The α-subunit contains a kinase domain, an autoinhibitory domain (AID), and a C-terminal ß-subunit binding domain [8]. The α subunit is primarily regulated by phosphorylation, which is essential for the enzymatic activity of AMPK [9,10,11,12]. The β subunit acts as a scaffolding component. It consists of a carbohydrate-binding module (CBM), which allows AMPK to bind glycogen, and a C-terminal domain that interacts with the α and γ subunits. The regulatory γ subunit contains four consecutive conserved cystathionine-β-synthase (CBS) domains, which sense cellular energy changes by binding AMP, ADP, or ATP, leading to AMPK activation through allosteric mechanisms and phosphorylation [8]. Each subunit has several isoforms (α1, α2, ß1, ß2, γ1, γ2, and γ3) encoded by distinct genes, resulting in up to 12 different possible AMPK isoforms. The different isoforms exhibit tissue-specific expression and regulatory properties. For example, α2 is primarily expressed in the cardiovascular system, especially in the heart, while α1 is more abundant in vascular endothelial cells [13,14]. AMPK is kept inactive by the AID’s interaction with the kinase domain, which is stabilized by ATP binding to the γ-subunit [15,16]. The schematic structure of AMPK is shown in Figure 1.

**Figure 2:**
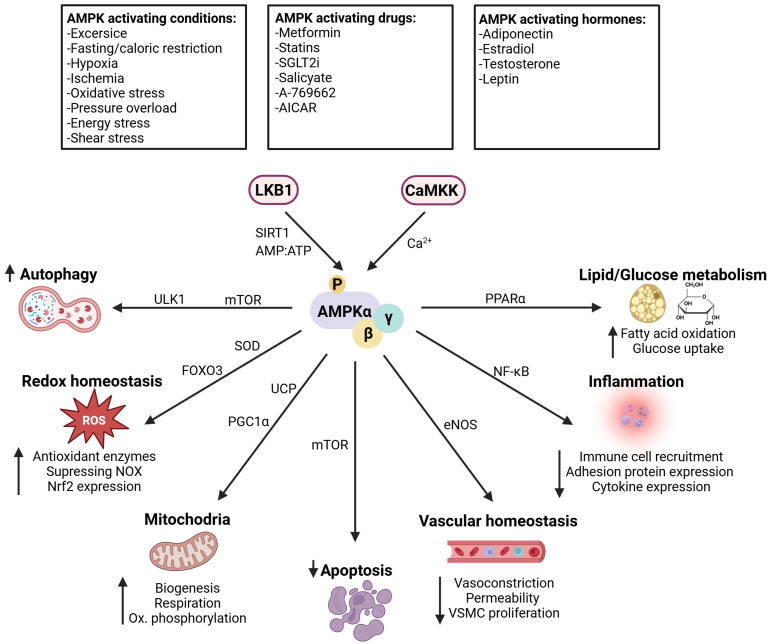
AMPK activation and downstream effects in cardiovascular diseases. Different conditions, drugs, or hormones lead to AMPK activation via LKB1 or CaMKK. Various pathways mediate the downstream effects of AMPK activation, leading to increased autophagy, expression of antioxidant enzymes, mitochondrial biogenesis, vasodilation, fatty acid oxidation, and glucose uptake, whereas apoptosis and inflammation are decreased overall. AICAR = 5-aminoimidazole-4-carboxamide riboside, LKB1 = liver kinase B1, CaMKK = calmodulin-dependent protein kinase kinase, SIRT1 = sirtuin 1, mTOR = mammalian target of rapamycin, SOD = superoxide dismutase, FOXO3 = forkhead box O3, UCP = uncoupling proteins, PGC1α = peroxisome proliferator-activated receptor gamma coactivator 1-alpha, eNOS = endothelial nitric oxide synthase, NF-κB = nuclear factor κ-light-chain-enhancer of activated B cells, peroxisome proliferator-activated receptor α, NOX = NADPH oxidase. The figure was created with BioRender.com.

AMP and adenosine diphosphate (ADP) binding leads to its activation through phosphorylation by liver kinase B1 (LKB1) and calmodulin-dependent protein kinase kinase β (CaMKKβ) [18,19], with recent evidence suggesting that increased intracellular calcium can also directly activate AMPK [20,21]. Different pharmacological and natural compounds are described as AMPK activators, which either directly bind to AMPK or indirectly lead to AMPK activation by increasing the AMP/ATP ratio or Ca^2+^ accumulation [8]. Biguanides, such as metformin, thiazolidinediones (TZDs), sodium–glucose cotransporter 2 inhibitors (SGLT2i), and statins, are indirect AMPK activators that mediate cardiovascular protective effects [22,23,24]. Furthermore, polyphenols, which are found naturally in substances such as resveratrol (red grapes), curcumin, genistein (soybeans), berberine, quercetin, or epigallocatechin gallate (green tea), can also lead to AMPK activation [8]. The powerful antioxidant α-Lipoic acid (ALA) also indirectly activates AMPK and was shown to have beneficial effects on cardiovascular diseases [25]. AMPK is directly activated by 5-aminoimidazole-4-carboxamide riboside (AICAR), which generates AMP-mimetic thienopyridone (A-769662) and benzimidazole (Compound 911) derivatives through allosteric AMPK activation and the prevention of dephosphorylation [8]. Further, salicylate (pro-drug of aspirin) shares structural similarities with A-769662 and activates AMPK similarly [26]. Compound-13, PT-1, and MT 63-78 (Debio0930) have also been identified in screenings as allosteric activators of AMPK [8]. In addition to the natural and synthetic AMPK activators, AMPK is also physiologically activated in response to hypoxia, ischemia, oxidative stress, pressure overload, shear stress, energy stress, exercise, and fasting or caloric restriction [17,27,28]. In general, metabolic pathways, such as lipogenesis (e.g., fatty acids, cholesterol), glycogenolysis, or protein synthesis, are negatively regulated by AMPK activation, whereas glucose uptake, glycolysis, fatty acid oxidation, mitochondrial biogenesis, and autophagy are positively regulated [8]. The downstream effects of AMPK activation, particularly in relation to cardiovascular diseases, are described in the following text and summarized in Figure 2.

## 2. Antioxidant Properties in Cardiovascular Disease

### 2.1. Antioxidant Properties

AMPK is an important cellular energy sensor essential for maintaining cellular homeostasis [29]. In addition to its metabolic regulatory function, AMPK is also involved in antioxidant responses. Oxidative stress, caused by the overproduction or insufficient breakdown of ROS or inadequate repair of oxidative damage [30], can activate AMPK [17]. AMPK then promotes multiple mechanisms that exert protective effects on various organ systems, including the cardiovascular system.

**Figure 3:**
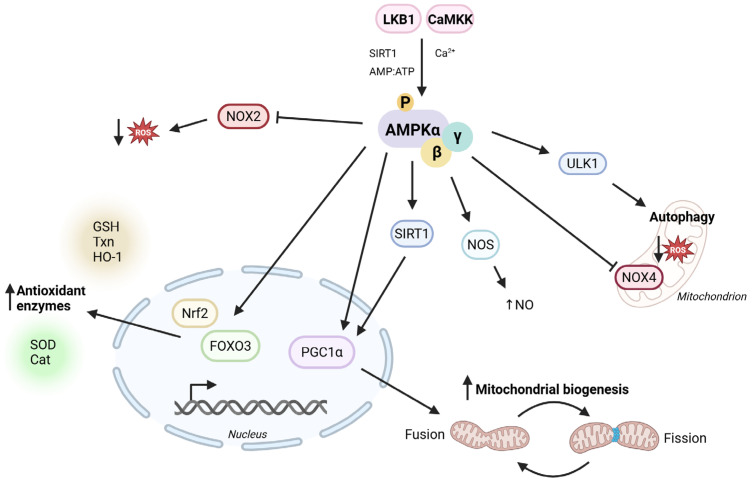
Antioxidant properties of AMPK. AMPK activation promotes various pathways involved in antioxidant defense of the organism, leading to increased autophagy, expression of antioxidant enzymes, and mitochondrial biogenesis and decreased reactive oxygen species production. LKB1 = liver kinase B1, CaMKK = calmodulin-dependent protein kinase kinase, SIRT1 = sirtuin 1, Nrf2 = nuclear erythroid 2-related factor 2, FOXO3 = forkhead box O3, PGC1α = peroxisome proliferator-activated receptor gamma coactivator 1-alpha, eNOS = endothelial nitric oxide synthase, NO = nitric oxide, NOX = NADPH oxidase, ULK1 = Unc-51-like kinase 1, ROS = reactive oxygen species, SOD = superoxide dismutase, Cat = catalase, GSH = glutathione, Txn = thioredoxin, HO-1 = heme oxygenase-1. The figure was created with BioRender.com.

One primary way AMPK mitigates oxidative stress is by suppressing NADPH oxidases (NOXs) [31,32], which are major contributors to ROS generation in the cardiovascular system [33]. Pharmacological AMPK activation has been shown to modulate NOX2 activity, leading to decreased superoxide production in various cell types, including immune cells [34], cardiomyocytes [35,36], and endothelial cells [37]. Thus, AMPK protects against oxidative stress-induced cell damage (Figure 3).

Furthermore, AMPK enhances mitochondrial biogenesis and autophagy. AMPK can directly interact with peroxisome proliferator-activated receptor gamma coactivator 1-alpha (PGC-1α) via phosphorylation [38]. PGC-1α is a transcriptional cofactor involved in the regulation of mitochondrial biogenesis, respiration, and oxidative phosphorylation [39]. The activation of PGC-1α via AMPK leads to improved mitochondrial function and reduced oxidative stress [40]. In addition, AMPK promotes PGC-1α expression through p38MAPK and sirtuin 1 (SIRT1) activation [41,42]. Simultaneously, AMPK stimulates autophagy by phosphorylating ULK1, facilitating the clearance of damaged mitochondria and thereby preventing excessive ROS accumulation [43,44].

Several studies have shown that AMPK can enhance the expression of antioxidants through the nuclear erythroid 2-related factor 2 (Nrf2) and SIRT1 pathways. Under normal conditions, Nrf2 is ubiquitinated by cullin 3 and Kelch-like ECH-associated protein 1 (Keap1) in the cytoplasm [45]. In cases of oxidative stress, Nrf2 is released and translocated into the nucleus, where it promotes the transcription of key components of the antioxidant defense system such as thioredoxins (Txn), glutathione (GSH) synthesizing and reducing enzymes, and heme oxygenase-1 (HO-1) [46]. Joo et al. were among the first to demonstrate that AMPK can phosphorylate serine residues to facilitate Nrf2 nuclear translocation [47]. Matzinger et al. linked the AMPK-induced Nrf2 phosphorylation to the degree of activation of its target genes [48]. Research has indicated that pharmacological activation of AMPK by AICAR increases HO-1 expression in an Nrf2-dependent manner in human endothelial cells [49]. AMPK activation also stimulates the SIRT-1-FOXO pathway [50]. Specifically, the activation of FOXO3 via SIRT-1 leads to enhanced expression of antioxidant enzymes, such as catalase and superoxide dismutase (SOD), further strengthening cellular defense mechanisms against oxidative stress [51].

By integrating these antioxidant functions, AMPK serves as a potent regulator of redox homeostasis, making it a promising therapeutic target for cardiovascular diseases characterized by oxidative stress.

### 2.2. AMPK and Improvement of Vascular Function

AMPK plays a crucial role in vascular function and disease by acting as a metabolic sensor that regulates endothelial function, inflammation, and oxidative stress. AMPK activation is linked to protection and supports the re-establishment of vascular function [52]. Dynamic changes in vascular biology drive the progression of cardiovascular disease, with the endothelium playing a crucial role in maintaining vascular function [53,54]. The inner lining of blood vessels regulates vascular tone, blood fluidity, inflammation, and immune responses through the secretion of various molecules [54,55]. The proper function of the endothelium primarily relies on the endothelial nitric oxide synthase (eNOS)-derived production of nitric oxide (NO), which is essential for endothelium-dependent vasodilation [56,57]. A decrease in NO bioavailability leads to endothelial dysfunction (ED), which fosters the development of cardiovascular diseases such as hypertension and atherosclerosis [58].

Physiological, pharmacological, and hormonal stimuli, including shear stress, statins, or adiponectin, can directly activate AMPK, leading to eNOS phosphorylation at Ser633/635 and increased NO production [59,60,61,62]. It has been shown that AMPK knockout or its pharmacological inhibition decreases this eNOS phosphorylation, thus limiting NO production [59]. Additionally, AMPK has been shown to cause an inhibitory phosphorylation of eNOS at Thr495 [63].

In recent years, new substances have been identified as mitigating endothelial dysfunction in an AMPK-dependent manner, including an indolic derivate IND6 [64] and ginsenoside RH4 [65].

In addition to being expressed in endothelial cells, AMPK is also found in vascular smooth muscle cells (VSMCs) [66,67]. Research has shown that AMPK contributes to endothelium-independent vasodilation in large and small arteries [66,67,68] and inhibits VSMC proliferation and inflammation [69]. Rodriguez et al. demonstrated that AMPK activation induces vasodilation by decreasing the intracellular Ca^2+^ concentration through its sequestration to the sarcoplasmic reticulum (SR), which is facilitated by increased SR calcium-ATPase (SERCA) activity in VSMCs [70]. In addition, other studies have shown that AMPK promotes relaxation through Ca^2+^-independent mechanisms and decreases Ca^2+^ sensitivity [71,72]. Furthermore, AMPK suppresses VSMC proliferation and migration [69,73] and inhibits vascular calcification [74].

The overproduction of ROS is a crucial pathogenic factor underlying endothelial and VSMC dysfunction in cardiovascular diseases [52]. The primary ROS sources in the vascular wall are mitochondria, NOX, xanthine oxidase, and uncoupled eNOS [75]. ROS generation is counterbalanced by antioxidants, including SOD, catalases, glutathione peroxidase, or peroxiredoxins [76]. AMPK activation by AICAR inhibited ROS production by promoting thioredoxin via the FOXO3 pathway in human endothelial cells [77] and reduced mitochondrial ROS by upregulating manganese SOD in human umbilical vein endothelial cells [78]. Furthermore, AMPK activation has been shown to reduce NOX4- and NOX2-derived ROS production in renal arteries [70]. Consistently, α1AMPK deletion led to an upregulation of vascular NOX2 [79]. Li et al. showed that AMPK activation ameliorates ED by suppressing mitochondrial ROS-associated endoplasmic reticulum stress and subsequent thioredoxin-interacting protein (TXNIP)/NLRP3 inflammasome activation [80].

AMPK activity mediates anti-inflammatory signaling and appears to link metabolic and inflammatory processes by controlling immune cell determination in response to tissue injury, which is energy-consuming [7,81]. For example, α1AMPK signaling is involved in mediating metabolic adaptation and adequate T-cell response during inflammatory processes [82,83]. Macrophages lacking α1AMPK are dysfunctional in maintaining the transition of pro-inflammatory M1 to anti-inflammatory M2 macrophages, resulting in impaired skeletal muscle regeneration after injury in mice [84]. Studies have shown that AMPK inhibits nuclear factor-kappa B (NF-κB), which is responsible for the expression of pro-inflammatory cytokines, including tumor necrosis factor-alpha (TNF-α) and interleukin 6 (IL-6). These cytokines, as well as adhesion molecules such as vascular cell adhesion molecule 1 (VCAM-1) or intracellular adhesion molecule 1 (ICAM-1) [85,86,87], are responsible for inflammatory responses and vascular immune cell infiltration [88].

### 2.3. AMPK and (Cardio)Vascular Diseases

In atherosclerosis pathogenesis, AMPK plays an important role by regulating autophagy, inflammation, and lipid metabolism in the endothelium. Atherosclerosis development is predominantly observed in regions with low shear stress, associated with decreased AMPK expression and increased mammalian target of rapamycin (mTOR) expression, leading to impaired autophagy [27,89]. The impaired autophagy leads to the development of an atherosclerotic phenotype, resulting in increased inflammation by translocating cell adhesion proteins to the outer cell membrane (e.g., ICAM-1), apoptosis, and senescence [89]. During atherosclerosis, a transformation of macrophages into foam cells is observed due to enhanced lipid uptake. In studies using bone marrow-derived macrophages, lipid dynamics and autophagy were found to be mediated by AMPK. However, AMPK signaling is impaired during foam cell formation, diminishing its protective effects against atherosclerosis despite initial activation by atherogenic lipoproteins [90]. AMPK dysregulation also increases cholesterol levels and monocyte mobilization, exacerbating atherosclerosis [91]. AMPK activation has anti-atherosclerotic effects by promoting cholesterol efflux through upregulating distinct transporters and inhibiting foam cell formation through mTOR pathway suppression, which reduces lipid accumulation in macrophages [92,93].

AMPK activation can ameliorate endothelial dysfunction by modulating inflammatory pathways and enhancing cellular defense mechanisms. Genetic α1AMPK deficiency promotes atherosclerotic vessel calcification, highlighting the protective role of AMPK [74]. Further, an endothelial–mesenchymal transition is typically observed during atherosclerosis development triggered by turbulent flow and endothelial ROS production. ROS production in the endothelial cells leads to an onset of mesenchymal gene expression, promoting the endothelial–mesenchymal transition [94]. Pharmacological activation of AMPK via AICAR has, in turn, a vasoprotective effect via the onset of endothelial gene expression instead of mesenchymal gene expression, which prevents endothelial–mesenchymal transition [94]. In coronary artery disease patients, specific microRNAs (miRNA-93 and miRNA-484) were identified to be upregulated, accompanied by decreased AMPK activation and eNOS inhibition [95]. AMPK activation seems promising in mitigating atherosclerosis, but further research must be performed to fully understand the potential therapeutic applications in cardiovascular disease.

AMPK dysregulation also plays a role in the pathophysiology of hypertension, as it is linked to lipid metabolism and inflammation. In spontaneous hypertensive rats, it was demonstrated that AICAR treatment and respective AMPK activation reduced ROS generation and inflammatory cytokine production. Further, sympathetic nerve activity was also reduced, leading to decreased blood pressure [96]. In addition, arterial hypertension in mice induced via angiotensin-II treatment also leads to endothelial dysfunction and a drastic increase in oxidative stress in the vascular wall. The hypertensive phenotype in these mice was attenuated by AMPK activation via AICAR treatment, leading to improved endothelial function and reduced oxidative stress in the vascular wall. In this study especially, α1AMPK was identified as a protective enzyme in the vascular system [97]. A follow-up study demonstrated that low-dose angiotensin-II infusion in α1AMPK knockout mice also led to endothelial dysfunction and oxidative stress in the vascular wall. In addition, increased NOX2 expression was observed, leading to endothelial inflammation [7]. Endothelial α1AMPK knockout mice demonstrated impaired endothelial cell barrier function associated with increased inflammatory cell recruitment into the vascular wall. In consequence, inflammatory proteins (e.g., CCR2, MCP-1), vascular adhesion proteins (e.g., VCAM-1), and cytokine expression, as well as NOX2 expression, were upregulated in the aorta of these mice [37]. Taken together, endothelial α1AMPK seems especially important in mediating vascular function.

**Figure 4:**
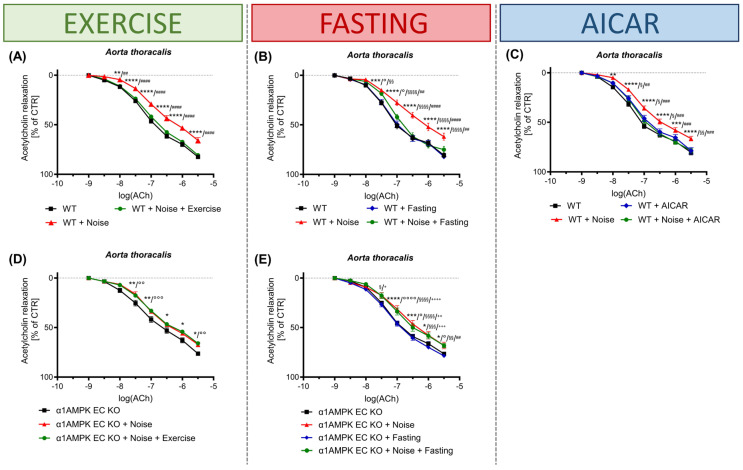
Effect of AMPK activation on negative effects of noise effects in vasculature. Exercise (**A**), caloric restriction (**B**), and AICAR (**C**) treatment prevent noise-induced endothelial dysfunction. On the other hand, genetic deletion of endothelial-specific AMPK abolishes the beneficial vascular effects of exercise (**D**) and fasting (**E**) in noise-exposed mice. *p*-values < 0.05 were considered significant. * = WT vs. Noise, # = WT + treatment vs. Noise, § = Noise vs. Noise + treatment, ° = treatment vs. Noise, + = treatment vs. Noise + treatment; treatment = exercise/fasting/AICAR. *, #, §, °, + *p* ≤ 0.05; **, ##, §§, °°, ++ *p* ≤ 0.01; ***, ###, §§§, °°°, +++ *p* ≤ 0.001; ****, ####, §§§§, °°°°, ++++ *p* ≤ 0.0001. Ach = acetylcholine, AICAR = 5-aminoimidazole-4-carboxamide riboside, WT = wild type, α1AMPK EC KO = endothelial-specific AMPK knock-out. The figure was reprinted from [28] with permission. Copyright © 2023, The Authors. Published by Oxford University Press. UK, on behalf of the European Society of Cardiology.

AMPK has also been shown to significantly influence vascular function in aircraft noise, a risk factor that has been increasingly better characterized in recent years [28]. It was shown that the activation of AMPK by intermittent fasting, voluntary exercise training, and AICAR normalized endothelial function. This protective modulation was abolished when AMPK was inactivated or downregulated at the endothelial level (Figure 4). By maintaining NO levels and reducing oxidative stress, inflammation, and immune cell infiltration, AMPK plays a pivotal role in sustaining endothelial function and preventing vascular disorders.

### 2.4. AMPK and Cardiac Function

Oxidative stress, mitochondrial dysfunction, and inflammation are among the key drivers of cardiac injury [37]. The role of AMPK in antioxidant defense, the inhibition of ROS production, and mitochondrial biogenesis is crucial for maintaining myocardial redox homeostasis and preserving myocardial integrity under stress conditions [98]. Mitochondrial dysfunction is believed to play an essential role in cardiac pathology. Due to their high energy demands, nearly one-third of cardiomyocytes are occupied by mitochondria, which generate over 95% of the ATP in the myocardium [99].

Apart from being the main energy source, mitochondria are also the primary source of ROS in cardiomyocytes [100]. Studies indicate that AMPK modulates the activity of uncoupling proteins (UCPs) via the peroxisome proliferator-activated receptor α (PPAR-α) axis [101,102]. The overexpression of UCP2 in neonatal rat cardiomyocytes protected the cells from oxidative stress-mediated damage [103], and activation of the AMPK/UCP2 pathway protected from indoxyl sulfate-induced cardiac hypertrophy and oxidative stress in isolated cardiomyocytes [104]. Additionally, it has been shown that UCP1 knockout worsens acute myocardial ischemia in rats [102]. Mitochondria are highly dynamic organelles continuously undergoing structural changes regulated by mitochondrial fusion and fission [105]. An imbalance in these dynamic processes results in mitochondrial dysfunction [106]. Du et al. showed that pharmacological AMPK activation mitigates myocardial ischemia–reperfusion injury mainly by inhibiting dynamin-1-like protein (Drp1)-mediated mitochondrial fission [107]. Especially under increased workload, AMPK is vital for mitochondrial function and influences energy substrate utilization and ATP production [108,109]. AMPK, specifically the α2 subunit, has been implicated in the induction of Bcl2-like protein 13-mediated mitophagy, resulting in cardioprotective effects [110].

Cardiac injury, often associated with oxidative stress, can cause structural changes in the heart, such as myocyte hypertrophy or fibrosis. These changes contribute to the development of cardiac dysfunction, resulting in heart failure [111]. AMPK acts as an energy sensor, enhancing ATP production in a failing heart by improving glucose metabolism, mitochondrial function, and fatty acid oxidation [112,113,114]. It is, however, also involved in other physiological processes essential for cardiac function. It has been shown that AMPK stimulation activates the mTORC1–autophagy cascade, suppressing cardiomyocyte enlargement and cardiac remodeling [115]. Moreover, AMPK activation by AICAR improved cardiac function in mice by inhibiting autophagy in failing hearts, possibly via the mTORC2 pathway [116].

AMPK also plays a vital role in maintaining cardiac function, particularly under stress conditions like myocardial infarction, by enhancing energy metabolism, protecting against cell death, and supporting mitochondrial function [17]. The heart is highly sensitive to changes in oxygen supply, and ischemia-induced hypoxia is one of the most potent physiological AMPK activators [117]. Hypoxia and peroxynitrite-derived ischemia–reperfusion (I/R) lead to the AMPK-mediated eNOS phosphorylation of Ser1177 and its activation [7]. AMPK activation before ischemia–reperfusion (I/R) injury by metformin in diabetic mice or A769662 in WT animals enhances eNOS phosphorylation and reduces infarct size and apoptosis [118,119]. AMPK activation also reduces the expression of inflammatory cytokines such as TNF-α, IL-6, and IL-1β, which are elevated during myocardial I/R injury [107].

After myocardial infarction, exercise induces AMPK phosphorylation and activation, which is linked to improved cardiac function by promoting myocardial repair and reducing complications like heart failure [120]. Additionally, natural antioxidants [121], melatonin [122], activated protein C [123], and antithrombin [124] mitigate I/R damage through AMPK activation by reducing oxidative stress and inflammation. In summary, AMPK activation is associated with protective effects during myocardial infarction and I/R injury; its dysregulation can lead to adverse outcomes, particularly in aging populations or those with metabolic disorders.

Pharmacological AMPK activation has been shown to improve cardiac function by reducing oxidative stress, inflammation, and apoptosis through the AMPK/SIRT1 signaling pathway [125,126]. Additionally, activating the AMPK/PPARα pathway enhances myocardial fatty acid metabolism, which decreases cardiomyocyte hypertrophy and fibrosis, thus alleviating the progression of heart failure [127]. Further, α2AMPK knockout mice showed impaired glucose uptake, glycolysis, and fatty acid oxidation during I/R, leading to increased apoptosis and reduced contractility [128]. AMPK activation through lifestyle interventions, including caloric restriction and exercise, has enhanced left ventricular function in failing hearts [113,114,120]. 

Long-term elevated blood pressure and pressure overload contribute to the development of cardiac hypertrophy and could lead to heart failure. AMPK activation is associated with attenuating the development of cardiac hypertrophy, for example, by inhibiting distinct protein synthesis [17]. Indeed, AMPK activation leads to the inhibition of key pro-hypertrophic signaling pathways like the nuclear factor of activated T cells (NFAT) transcription factor expression and the mitogen-activated protein kinases (MAPK) ERK1/2 [129]. In addition, AMPK further reduces protein synthesis by inhibiting mTOR signaling, which is critical for hypertrophic growth [130]. Transverse aortic constriction (TAC) in α2AMPK-deficient mice leads to an exacerbation of pressure overload-induced left ventricle hypertrophy, indicating a protective effect of α2AMPK expression [131]. In experimental models, AMPK activation via AICAR, metformin, or adiponectin has effectively reduced pressure overload and prevented cardiac hypertrophy [132,133,134,135]. Oxidative stress is also associated with cardiac hypertrophy progression, which is inhibited by activating the LKB1/AMPK axis by preventing abnormal cardiomyocyte growth [136]. Further ROS production is inhibited via AMPK-mediated activation of antioxidant enzymes like thioredoxin-1 and SOD2 [137]. Autophagy is also regulated by AMPK, with dual effects on one side, promoting autophagy and leading to the clearance of damaged proteins. In contrast, on the other side, excessive autophagy may cause heart failure. It was demonstrated in a chronic model of heart failure that AMPK activation was associated with decreased autophagy levels, leading to improved cardiac function [116]. Cardiac energy metabolism is shifted via AMPK towards fatty acid oxidation, which is beneficial under stress conditions [132]. Further, AMPK affects cytoskeletal remodeling, which is essential for maintaining cardiac structure and function during hypertrophic responses [132]. Lastly, the post-translational modification of serine and threonine residues by O-GlcNAcylation is associated with cardiac hypertrophy and increased workload, which was attenuated by AMPK, preventing cardiac hypertrophy [138].

In summary, AMPK is essential in myocardial redox regulation, mitochondrial protection, and heart failure protection. Its involvement in various cellular pathways, including mitochondrial dynamics, autophagy, inflammation, and antioxidant defense, underlines its potential as a therapeutic target in cardiovascular diseases.

## 3. Sex-Specific Differences of AMPK Regulation in Cardiovascular Diseases

### 3.1. Sex-Specific Regulation of AMPK Activity

AMPK exhibits sex-specific properties regarding its activation, regulation, and downstream effects. The complex interplay of sex hormones, sex chromosomes, and metabolic differences influences AMPK activity and contributes to distinct metabolic profiles and health outcomes [139].

In particular, sex hormones have been shown to modulate AMPK activity. The female-related hormone estrogen activates AMPK via several mechanisms [139]. In cardiac tissue, estrogen signaling can modulate voltage-gated Ca^2+^ channels, leading under physiological conditions to vasodilation and immune cell signaling, whereas under pathological conditions, estradiol signaling leads to an exacerbation of cardiovascular diseases [140]. In human endothelial cells, estradiol stimulation activates AMPK via estrogen receptor β in a Ca^2+^-induced and CaMKK-dependent manner. The estradiol stimulation further leads to ACC and eNOS activation, which are primary targets of AMPK [141]. Estradiol-induced eNOS activation by AMPK rapidly increases coronary blood flow, which is associated with cardioprotective effects [142,143]. Further, it was shown in human aortic endothelial cells that estrogens mediate SIRT-1 expression [144], which leads to AMPK activation via SIRT-1-dependent LKB1 deacylation [145]. Estrogens mediate protection against ischemic brain injury by preventing neuronal death via activating the SIRT-1-dependent AMPK pathway during ischemic stroke events [146]. Estrogen-mediated signaling prevents hypertension-induced vascular damage [147], mediates anti-atherogenic effects, and reduces inflammation by preventing abnormal monocyte adhesion and transmigration into the vascular [148]. Overall, estrogens play an essential role in vascular and cardiac physiology and in protecting against cardiovascular diseases, probably mainly in an AMPK-mediated manner [139].

The primary male sex hormone testosterone and androgens, which males and females produce, also appear to have different effects on AMPK activity [149]. The interplay of testosterone levels and AMPK activity is particularly relevant in aging men, where low testosterone correlates with decreased AMPK activity, which is associated with increased cardiovascular risk and metabolic disorders [149,150]. Like estrogens, androgens activate AMPK in a Ca^2+^-induced CAMKK2-dependent manner and by LKB1 phosphorylation [151,152]. Testosterone-enhanced AMPK activity promotes glucose metabolism and fatty acid oxidation, vital for anabolic functions like energy homeostasis in cardiomyocytes, muscle mass increase, and bone density [149,150]. Testosterone deficiency was identified as a risk factor associated with higher mortality rates, for example, in type 2 diabetes mellitus, hypertension, or stroke [153]. In the literature, it is controversially discussed whether testosterone replacement therapy improves cardiovascular outcomes in men with low testosterone by enhancing vascular function and exercise capacity [154]. Conversely, excessive levels of abuse of anabolic steroids lead to adverse cardiovascular events like impaired vascular function [139,155]. The protective effects of testosterone seem to be concentration-dependent. Still, the exact mechanism of testosterone affecting AMPK in cardiovascular diseases is poorly understood and needs further investigation.

### 3.2. Sex-Specific Effects of AMPK Activity on Cardiovascular Diseases

**Figure 5:**
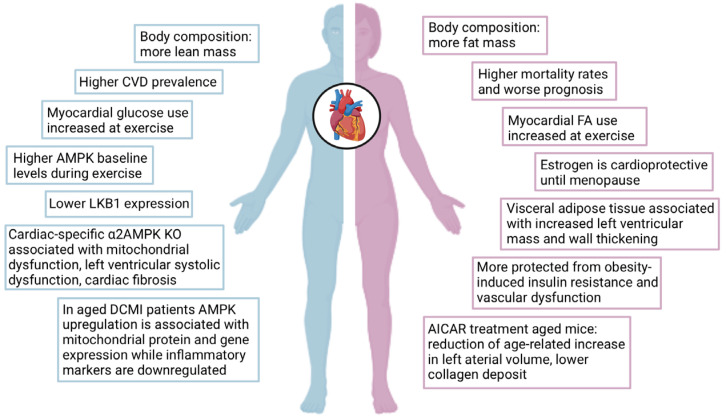
Impact of sex and AMPK activity on the cardiovascular disease development. AMPK activity has sex-specific effects in the development and progression of cardiovascular diseases. CVD = cardiovascular disease, LKB1 = liver kinase B1, KO = knockout, DCMI = dilated cardiomyopathy, FA = fatty acid, AICAR = 5-aminoimidazole-4-carboxamide riboside. The figure was created with BioRender.com.

Women have a lower prevalence of cardiovascular diseases than men. Still, after acute cardiovascular events, women have a higher mortality rate and a worse prognosis, which is presumably caused by differences in disease symptoms or the perception of these symptoms by physicians, epidemiology, pathophysiology, environmental factors, gene expression, and sexual hormones [156,157]. For example, estrogen production is significantly reduced in menopausal women, which is associated with a higher risk of cardiovascular disease development compared to men of the same age [158], which has been attributed to a decline of estrogen-dependent AMPK activation [159]. Interestingly, estradiol increased phosphorylated AMPK protein expression in early postmenopausal women, whereas diminished AMPK phosphorylation was observed in late postmenopausal women [160]. It is not well understood what role testosterone replacement therapy would have in old men, since it should have an activating effect on AMPK [139], but on the other hand, there are well-known opposing effects of estrogen and testosterone on chronic cardiac remodeling and function in mice with myocardial infarction [161]. A summary of sex-specific effects of AMPK activity on cardiovascular diseases is shown in Figure 5.

AMPK, and its regulatory role in energy metabolism, is probably also responsible for sex-specific differences in overall body composition and the metabolic profile of the heart. In general, the female body consists of more fat mass than lean mass compared to males [162]. A UK Biobank cohort study demonstrated that visceral adipose tissue in women but not in men was associated with increased left ventricular mass and wall thickening [163]. In both sexes, higher muscle mass is associated with a lower risk of cardiovascular diseases and mortality. Still, in another multicenter study regarding whether differences in body composition contribute to sex-specific differences in cardiovascular disease mortality, it was demonstrated that in women, independent of the muscle mass level, high-fat was associated with higher mortality. This highlights the importance of increasing activity and improving muscle mass to reduce cardiovascular risk [164]. In response to exercise and metabolic conditions, researchers indicate that AMPK activation is generally higher in males than females. During submaximal exercise, men showed significantly higher skeletal muscle activation of α2AMPK, lower energy charge, and fat oxidation than women [165]. In an exhaustive exercise test, male mice demonstrated a more robust cardiac AMPK activation than female mice [166]. The higher lipid oxidation in women during exercise is associated with lower cellular energy stress. Further, women seem to have a higher proportion of type 1 muscle fibers, which may also contribute to their metabolic differences [167]. Sex-dependent variations are also observed in LKB1 expression to be lower in males. In addition, AMPK activation in mice was higher in oxidative muscles than in glycolytic muscles, influencing the muscle phenotype and leading toward a slower oxidative profile [168].

**Figure 6:**
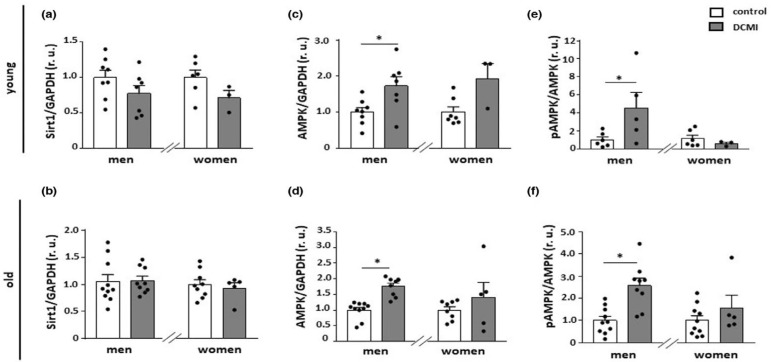
Changes in Sirt1 and AMPK expression. The comparison of Sirt1 (**a**,**b**), AMPK (**c**,**d**), and phosphorylated AMPK (**e**,**f**) expression in healthy and diseased (inflammatory dilated cardiomyopathy) males and females. Significantly upregulated expression and elevated phosphorylation of AMPK were observed in young and old males suffering from DCMI compared to non-diseased individuals. In females, no significant changes were observed. Data are shown as mean ± SEM (n = 3–10 per/group). * *p* < 0.05 vs. corresponding control. Data are normalized to the corresponding control and expressed in relative units. DCMI = inflammatory dilated cardiomyopathy, AMPK = AMPK-activated protein kinase, pAMPK = phosphorylated AMPK, GAPDH = glycerinaldehyde-3-phosphate-dehydrogenase, Sirt1 = sirtuin 1. Figure was reprinted from [171] with permission. © 2023 The Authors. Aging Cell published by Anatomical Society and John Wiley & Sons Ltd.

High fat diet (HFD)-treated female mice were protected from insulin resistance, hepatic steatosis, oxidative stress, and lipotoxicity compared to HFD-treated male mice, which were associated with adiponectin and AMPK signaling. These data indicate that females may have a protective mechanism against obesity-induced metabolic dysfunction related to AMPK signaling [169]. While males generally exhibit higher baseline AMPK levels, for example, during exercise, females may benefit from protective mechanisms that enhance AMPK signaling under certain conditions, such as obesity. The metabolic regulation of AMPK is complex, and more research has to be done in this direction. Further, cardiac-specific α2AMPK-deficient mice exhibit mitochondrial dysfunction associated with progressive left ventricular systolic dysfunction and cardiac fibrosis, particularly in males, again indicating a sex-specific response to AMPK signaling [109]. In aged mice (21 months), AICAR treatment reduced the age-related increase in left arterial volume in females, linked to lower collagen deposit and decreased Gil1 expression in female cardiac fibroblasts. Meanwhile, in aged male mice, AICAR had only minimal effects on male hearts due to blunted AMPK phosphorylation, resulting in no significant improvement in cardiac function [170]. In inflammatory dilated cardiomyopathy (DCMI) patients, cardiac AMPK expression and phosphorylation were significantly increased in male patients compared to females (Figure 6). Further, in aged male DCMI patients, AMPK was upregulated and associated with mitochondrial protein and gene expression, while in older females, oxidative phosphorylation genes were reduced. In addition, inflammatory markers like IL-18 expression were increased in older females, whereas at the same time, in male DCMI patients, inflammatory markers like NF-κB and TLR4 were downregulated [171,172].

Aged hypertensive rats developed heart failure associated with sex-specific cardiac remodeling. It was shown that male mice had a higher mortality at 1 year compared to females and WT males. The diastolic dysfunction was present in both sexes, while the left ventricular ejection fraction was only reduced in male rats. However, LV hypertrophy and reduced AMPK activity were specific to male hypertensive rats [173]. In conclusion, AMPK plays a central role in regulating energy metabolism in both males and females. Still, its activity and effects can differ between sexes due to the influence of sex-specific hormones, body composition, and other factors. Understanding these sex-specific differences in AMPK regulation is essential for developing and optimizing therapeutic strategies for metabolic diseases based on an individual’s sex.

## 4. Clinical Implication of AMPK

As already shown in Figure 2, several drugs with AMPK-activating properties have become indispensable in medicine. Thus, metformin mediates an inhibition of gluconeogenesis and fatty acid synthesis and increases glucose uptake through AMPK activation [174]. This improvement in glucose metabolism makes metformin one of the most essential drugs in treating type 2 diabetes mellitus [175,176]. Sodium–glucose co-transport 2 inhibitors (SGLT2i) and Glucagon-like peptide-1 receptor agonists (GLP1-RAs) have similar AMPK-mediated properties to metformin. However, in addition to the primary influence on glucose metabolism, the anti-inflammatory and antioxidant properties, as well as the improvement of vascular function of AMPK, come into play here [177]. Accordingly, these substances are also used in clinical practice to treat renal and cardiac failure beyond diabetes mellitus [178,179,180]. Another group of substances with AMPK-modulatory properties is the HMG-CoA reductase inhibitors, also called statins, whose effect is particularly based on the vascular protective and anti-inflammatory properties of AMPK [23]. As a result, statins are used in clinical routine for all forms of vascular diseases such as coronary heart disease, peripheral arterial disease, and cerebral arterial occlusive disease [181,182,183]. The drugs mentioned do not act on AMPK in isolation but also via other signaling pathways. Nevertheless, targeted AMPK activation with the help of AICAR also increased physical performance in animal models, which resulted in AICAR being listed by the World Anti-Doping Agency (WADA) [184].

Few gender-specific differences are still considered in clinical practice. Further focused studies are urgently needed to improve patient care through individualized medicine.

## 5. Conclusions

Cardiovascular diseases (CVDs) are a leading cause of morbidity and mortality worldwide, with a wide array of established treatment options. However, optimizing medical care requires individualized approaches that consider factors such as sex, age, and metabolic status. Understanding the molecular mechanisms driving CVDs is crucial for the advancement of next-generation therapies. One such mechanism involves AMP-activated protein kinase (AMPK), which plays a vital role in cardiometabolic regulation by reducing oxidative stress and enhancing cellular resilience. Despite its recognized importance, much of our knowledge about AMPK is derived from cell or animal models, underscoring the need for translational research to refine therapeutic strategies.

AMPK activation enhances antioxidant defenses, reduces mitochondrial dysfunction, and suppresses inflammatory pathways, collectively diminishing oxidative damage in vascular tissues. Notably, sex-specific differences in AMPK activity, often linked to hormonal influences such as estrogen, result in varying degrees of cardioprotection. These differences may help to explain the observed disparities in CVD susceptibility and progression between sexes. Understanding how AMPK signaling diverges across biological sexes is essential for guiding future research and developing sex-informed interventions. Translating these insights into clinical practice could improve the prevention and treatment of CVD risk factors like diabetes, hypertension, obesity, and hyperlipoproteinemia, as well as conditions such as heart failure, stroke, and coronary artery disease. AMPK represents a promising target for individualized therapies that integrate metabolic and hormonal influences to lessen the burden of cardiovascular diseases globally.
